# Nursing Program Promoting the Activity of Daily Living Among Older Adults Admitted to Semimedical Intensive Care Units: A Quasi-Experimental Study

**DOI:** 10.1155/nrp/3848134

**Published:** 2025-11-05

**Authors:** Chakkarin Sommana, Samoraphop Banharak, Panita Limpawattana, Supin Sim-Im, Porntip Pimpun, Teerawat Somkamsri, Orada Seeharach, Somphorn Chaisomsee, Walailuk Donsrichan, Boonnada Luejunda, Sirapath Navanukul

**Affiliations:** ^1^Department of Gerontological Nursing, Faculty of Nursing, Khon Kaen University, Khon Kaen, Thailand; ^2^Department of Internal Medicine, Faculty of Medicine, Khon Kaen University, Khon Kaen, Thailand; ^3^Nursing Department, Srinagarind Hospital, Khon Kaen University, Khon Kaen, Thailand

**Keywords:** critical care, disability, fundamental care, gerontology, older people, rehabilitation

## Abstract

**Aim:**

To investigate the effects of a nursing program to early promote the activity of daily living after passing critical conditions among older adults admitted to the semimedical intensive care unit.

**Design:**

Quasi-experimental research.

**Methods:**

A total of 50 older adults admitted to the semimedical intensive care unit were recruited using a consecutive sampling method. Data were collected from June 28 to October 3, 2024, using the Barthel ADL Index, complication record forms, and muscle strength record forms.

**Results:**

The findings indicated that the experimental group reported higher scores in performing activities of daily living (17.48 ± 1.19 vs. 2.76 ± 0.44) and motor power (4.96 ± 0.20 vs. 3.68 ± 0.48), as well as a lower incidence of complications (12% vs. 64%) than the control group (*p* < 0.001).

**Conclusion:**

The study demonstrates that the nursing program is effective in facilitating early functional recovery and reducing complications among older adults following critical conditions in the semi-intensive care unit. This program provides a valuable approach to enhancing the quality of care and outcomes for this vulnerable population.

**Trial Registration:**

Thai Clinical Trials Registry (TCTR): TCTR20240708010

**Summary**

• Impact
◦ Frailty, characterized by a decline in the ability to perform activities of daily living, affects older adults physically, mentally, socially, and economically, while also placing a burden on the healthcare system.
◦ This issue is particularly evident among older adults admitted to the intensive or semi-intensive care units, as many experience further loss of functional ability after transfer to another ward or discharge home.
◦ Therefore, nursing interventions that promote the recovery of activities of daily living early after stabilization of critical conditions are essential and should be implemented promptly.
• Patient and public contribution
◦ This study included older adults admitted to the semimedical intensive care unit.
◦ A panel of five experts, consisting of a physician specializing in critical care medicine, a physical therapist, a geriatric nursing instructor, and two registered nurse, was involved in verifying the content validity and ensuring consistency.

## 1. Background

Effective critical care management, including the use of mechanical ventilation, has substantially improved the survival rates among critically ill patients [1], particularly older adults, of whom approximately 20%–50% are admitted to the critical care units [2]. However, prolonged mechanical ventilation increases the risk of complications associated with ventilator-related pathologies, underlying comorbidities, adverse drug effects, and extended bed rest. These factors contribute to both physical and psychological impairments, most notably muscle weakness and joint contractures, as prolonged inactivity results in a loss of motor power of 1%–1.5% per day or 10%–15% per week [3, 4]. Consequently, critically ill patients often develop musculoskeletal dysfunction, which delays recovery, prolongs hospital stays, and increases ventilator dependency by two- to fivefold [4].

In older adults, immobility exacerbates health risks by causing muscle atrophy, reduced limb and respiratory muscle strength, and restricted joint mobility, all of which impair the ability to perform activities of daily living (ADLs) [5]. Older adults admitted to intensive (ICU) or semi-intensive care units (SICU), a step-down setting that provides close monitoring and specialized nursing care, are particularly vulnerable to neuromuscular disorders and muscle weakness [6, 7]. Evidence shows that critically ill patients who remain immobile for 5–7 days may experience up to a 50% decline in neuromuscular function [4, 5, 8, 9]. Moreover, more than five million older adults discharged from ICUs continue to experience persistent fatigue, weakness, and reduced mobility, which further compromise ADLs, diminish self-care capacity, and substantially lower overall quality of life [4, 5, 10].

The ability to perform ADLs is a key indicator of an older adult's capacity for independent living. ADLs include basic self-care tasks such as grooming, toileting, bathing, dressing, and eating [11]. Although hospitals may differ in when they assess ADLs, whether before admission, during treatment, or prior to discharge, the primary objective of healthcare professionals is to support older adults in maintaining independence [12]. For nurses, assessing and promoting ADLs are essential to help older adults recover to their pretreatment condition, or as close to it as possible. ADL performance also reflects the quality of care provided and serves as a critical basis for discharge planning and continuity of care.

A growing body of evidence shows that older adults experience a marked decline in their ability to perform ADLs compared to preadmission levels [13]. This decline often persists after discharge, with older adults facing ongoing challenges in rehabilitation and recovery, and is more pronounced than in younger adults [13, 14]. One study reported an ADL score of 98% prior to hospitalization, which dropped to 43.3% in the ICU, 51.7% before discharge, 73.89% at 1 month after discharge, 82.42% at 3 months, and 84.23% at 6 months [13]. Despite gradual improvement, many older adults continued to have limited self-care ability, with complications occurring in 37% of cases [15], including pressure ulcers, deep vein thrombosis, lower-extremity muscle atrophy, and reduced cardiopulmonary function. Such impairments not only compromise physical, mental, and social well-being but also place a considerable burden on caregivers and increase healthcare costs. Moreover, ADL performance in older adults has been identified as a significant health indicator, capable of predicting mild cognitive impairment, dementia, and mortality [13].

Based on clinical experience in the SICU, more than 60% of admitted patients are older adults. Upon transfer or discharge, many remain unable to care for themselves, relying on disposable diapers, remaining bedridden, and developing pressure sores. They require caregiver assistance for basic tasks such as repositioning, which places a considerable burden on families and increases economic costs. A review of admissions between 2019 and 2022 in the SICU of a super-tertiary university hospital in northeastern Thailand revealed that over 80% of patients were classified as totally dependent according to the Barthel ADL Index. Before transfer or discharge, the mean Barthel ADL score was 40.19 ± 5.33, with common complications including pressure sores and incontinence-associated dermatitis [16]. A review of the nursing process further indicated that healthcare personnel primarily prioritize acute treatment of critically ill patients, with limited emphasis on ADL enhancement or rehabilitation to prepare older adults for a safe return home.

A review of the national and international literature revealed a lack of specific programs to promote ADL among older adults. No comprehensive programs address all 10 dimensions of the Barthel ADL Index. Previous studies primarily focused on exercise for critically ill patients, with most samples consisting of adults. In addition, there were no established guidelines or programs within the ward aimed at promoting or restoring the ability to perform ADL among older adults. As a result, the research team was interested in examining the effects of a nursing program to early promote the ADL after passing critical conditions among older adults admitted to the SICU to prepare them for discharge or transfer out of the ward.

## 2. Aims

This study aims to evaluate the effects of a nursing program to promote early ADLs after passing critical conditions among older adults admitted to the SICU. It will focus on the Barthel ADL Index, complications, and motor power during hospital stay in the SICU between the control group and the experimental group.

## 3. Methods

### 3.1. Study Design

This study employs a quasi-experimental research design using a two-group pretest–posttest approach. The objective is to compare overall and item-specific Barthel ADL Index scores between a group receiving the ADL promotion program after recovering from a critical illness and a group receiving standard nursing care. In addition, the study assesses the incidence of complications and muscle strength throughout the intervention period, comparing findings between the experimental and control groups.

### 3.2. Setting

The setting of this study was the SICU at a super-tertiary university hospital in northeastern Thailand. The SICU consisted of three similar units caring for critically ill patients who were stepping down from ventilator support or partially recovering from critical conditions. These patients required continuous observation, with a nurse-to-patient ratio of 1:2. The total capacity of the SICU was 25 beds, with 7–8 beds per unit.

### 3.3. Sample Size Calculation

The sample size was calculated using the formula for comparing the means of two independent groups in experimental research [17]. A two-tailed test was applied with a significance level of 0.05 (*Z*_*α*_ = *Z*_0.05_ 1.96) and a power of 0.80 (*Z*_*β*_ = *Z*_0.1_ = 0.84). The calculation was based on the study by Ichikawa et al. [18], which reported the Barthel ADL Index as 86.3 ± 11.3 in the intervention group and 75.0 ± 15.0 in the control group. Substituting these values into the formula yielded a minimum required sample size of 22 participants per group. To account for a potential 10% dropout rate, as recommended by Grove et al. [19], the final sample size was adjusted to 50 participants, with 25 allocated to the experimental group and 25 to the control group. To prevent contamination between groups, data were first collected from the control group receiving standard nursing care until the last participant was enrolled, followed by enrollment of the experimental group for the nursing interventions.

### 3.4. Sample Selection

Participants were selected using consecutive sampling and were screened based on inclusion and exclusion criteria.

#### 3.4.1. Inclusion Criteria

1. Older adults aged ≥ 60 years, admitted to the SICU from the emergency department or other wards.
2. Patients or their legally authorized representatives who provided informed consent.
3. Clinically stable patients who had recovered from critical illness, free from septic shock, extubated ≥ 24 h, and hemodynamically stable.
4. Fully conscious, Thai-speaking patients deemed eligible for the intervention by their physician.

#### 3.4.2. Exclusion Criteria

1. Patients receiving palliative care in the terminal stage.
2. Patients who were bedridden prior to hospital admission.
3. Patients with neurological or neuromuscular conditions (e.g., myasthenia gravis, Guillain–Barre syndrome, and acute stroke), fractures, confusion, agitation, or muscle weakness.
4. Patients with preexisting chewing or swallowing impairments.

#### 3.4.3. Withdrawal Criteria

**Figure 1:**
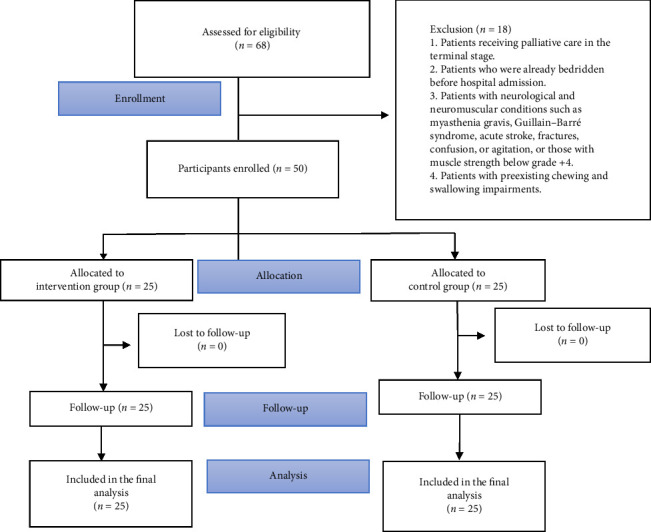
Consort diagram.

1. Patients who voluntarily withdrew.
2. Patients who left the unit before study completion.
3. Patients who passed away during the study period. All participants included in the study are presented in the CONSORT diagram (Figure 1).

## 4. Intervention

The nursing program consists of two main components as follows.

### 4.1. Nursing Intervention Based on the Barthel ADL Index

**Figure 2:**
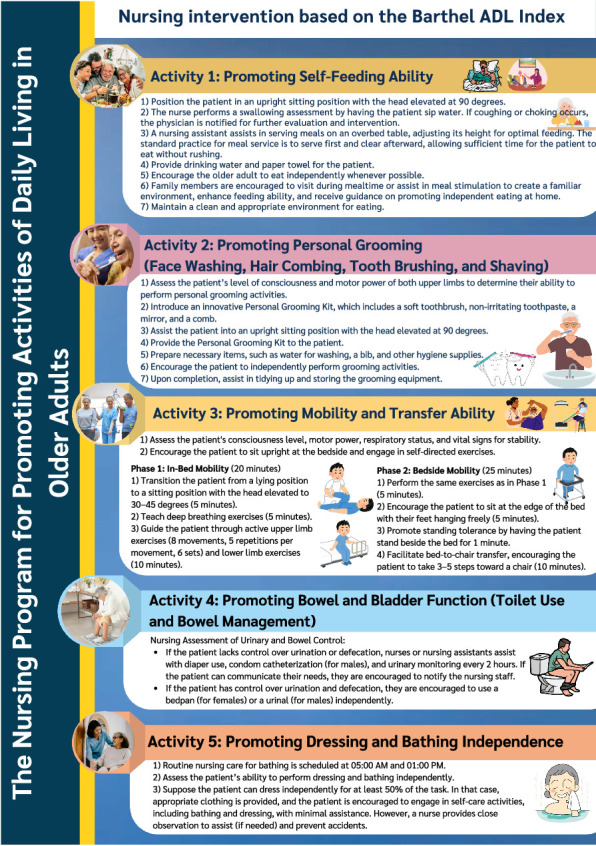
Nursing intervention based on the Barthel ADL Index.

This component is tailored according to patients' ADL capabilities and encompasses five key activities. Family members are actively involved in supporting and promoting ADL performance among older adults (Figure 2). They were educated about the program details and trained on how to create a supportive environment at the patient's bedside, as well as how to assist with feeding and encourage patients to perform ADLs.

### 4.2. Nursing Intervention Based on Nursing Daily Activities

**Figure 3:**
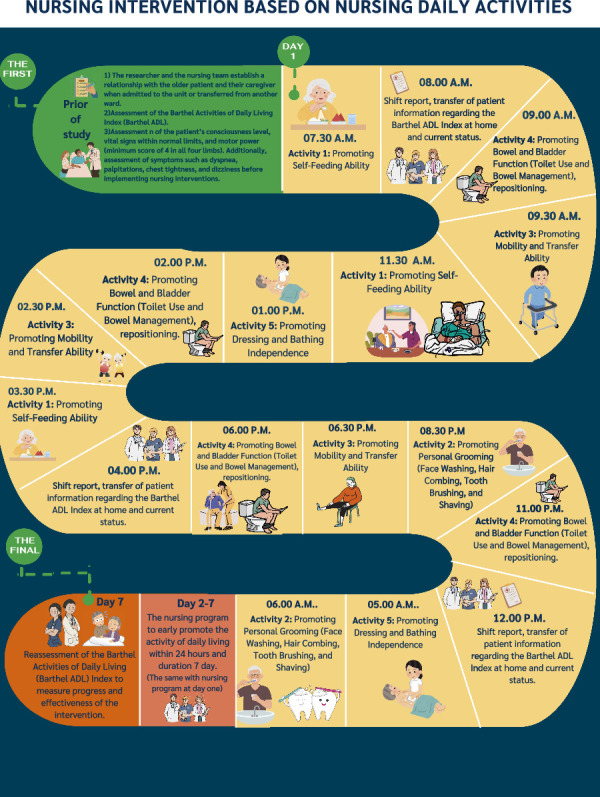
Nursing intervention based on nursing daily activities.

This component focuses on facilitating the performance of daily activities in older adults after stabilization from critical illness in the SICU. The program translates the five key ADL activities into structured daily tasks, providing explicit guidance for nurses and researchers on specific actions to be implemented over a 7-day period, with the duration and follow-up schedule tailored individually for each patient (Figure 3).

## 5. Data Collection Instruments

The data collection instruments consist of three sections as follows:

**Part 1:** General information record form comprising nine items: sex, age, BMI, occupation, cause of admission, APACHE II score, comorbidities, nutritional risk screening using the SPENT, and nutrition status by Nutrition Alert Form (NAF).
**Part 2:** ADL in older adults, recording complications during program implementation, and program monitoring.
    2.1. The Barthel Index for ADLs.
    The Barthel ADL Index assesses the patient's ability to perform basic daily tasks. The assessment consists of 10 items (feeding, grooming, transfer, toilet use, mobility, dressing, stair climbing, nothing, bowel control, and bladder control), with a total score ranging from 0 to 20. Higher scores indicate greater independence in performing ADLs [20].
    2.2. Motor power record form.
    A medical doctor assessed motor power in all four extremities, right and left arms and right and left legs. A four-level scale indicated the strength of the muscles in each limb, with higher levels representing greater muscle power. The results were then recorded in a standardized form for data analysis.
    2.3. Recording complications during program implementation.
    A complication recording form was developed to document various types of complications, including acute confusion (delirium), respiratory failure, infections (sepsis), reintubation, and other relevant conditions. These complications were diagnosed by physicians and documented in the medical charts. The outcomes were subsequently extracted from the medical records. The number of occurrences for each type of complication and the total number were recorded for final comparison.
    2.4. Types of oxygen support.
    An oxygen support classification form was developed, consisting of a single item with multiple response options: room air, nasal cannula, high-flow nasal cannula (HFNC), noninvasive ventilation (NIV), and endotracheal tube (ET-tube). This form was used for final comparison, with more advanced oxygen support indicating greater patient severity.
**Part 3:** Program implementation checklist (activities' checklist): This section consists of five items as follows: Activity 1: Ability to eat (feeding); Activity 2: Ability to wash face, comb hair, brush teeth, and shave (grooming); Activity 3: Ability to transfer from bed to chair or sit up from bed (transfer), move around the room or home (mobility), and go up and down one flight of stairs (stair); Activity 4: Ability to use the toilet (toilet use) and control bowel and bladder function (bowel); and Activity 5: Ability to dress (dressing) and bathe (bathing). The head nurse monitors the program's implementation and records the evaluation results.

## 6. Data Collection Procedure

The study was conducted in two phases as follows:

6.1. Instrument development phase: The nursing intervention program was developed based on a systematic literature review by Worraphan et al. [21] and structured into three components: (1) a nursing program based on the Barthel ADL index, (2) a nursing program based on the daily nursing activities, and (3) data collection instruments comprising three sections.
6.2. Instrument validation and implementation: Five experts, including a geriatric internal medicine specialist, a physical therapist, a nursing lecturer, and two registered nurses, assessed the program's validity. We calculated the content validity index (CVI) and item–objective congruence index (IOC).
    6.2.1. A nursing program based on the Barthel ADL index had CVI = 0.95 and IOC = 0.85
    6.2.2. A nursing program based on the daily nursing activities had CVI = 1.00 and IOC = 0.90
    6.2.3. The data collection instruments consist of three sections that had CVI = 1.00 and IOC = 0.90
    6.2.4. Barthel ADL assessment had interobserver reliability = 0.71 and intraobserver reliability = 0.97 [20].

## 7. Ethical Considerations

This study was approved by the Human Research Ethics Committee of Khon Kaen University (Approval No. HE 671105; dated: June 28, 2024). Participants and their legally authorized representatives were fully informed about the study objectives, procedures, data collection timeline, anticipated benefits, additional monitoring measures, safeguards for cognitive decline, and their right to withdraw at any time without affecting medical care. All data were handled confidentially, and results were reported in aggregate form. Older adults and their family members who were interested in this study signed the informed consent before we started conducting this study.

## 8. Data Collection Process

After protocol registration and ethical approval, the ward administrator received a letter requesting permission to collect data. The head nurse and registered nurses met to discuss the data-gathering procedure to ensure mutual understanding and compliance. The nurses in charge of the samples' care received training in the program and finished it after 7 days of participation. A research assistant who was not involved in the day-to-day operations of the ward conducted the initial assessments. All five of the program's activities were also given to the samples. The lead nurse used a checklist to evaluate the progress. Finally, the research assistant evaluated the outcomes of this study.

## 9. Data Analysis

Data were analyzed using SPSS Version 29.0.1.0 (licensed by Khon Kaen University). Normality was assessed using the Kolmogorov–Smirnov test, with skewness and kurtosis statistics examined to evaluate the distribution characteristics. When normality assumptions were not met, nonparametric tests were applied instead of parametric methods. Parametric statistics were used for data analysis as follows: (1) Descriptive statistics: General sample characteristics were analyzed using percentages, means, standard deviations, medians, and minimum and maximum values. Differences in categorical variables were tested using the chi-square or Fisher's exact test, while continuous variables were compared using an independent *t*-test. (2) Within-group ADL score analysis: Pre- and postintervention ADL scores were compared using a Wilcoxon signed rank test. (3) Between-group ADL score comparison: Control and experimental groups were compared using a Mann–Whitney *U* test. (4) The comparison of motor power before and after applying nursing programs between group comparisons: Control and experimental groups were compared using a Mann–Whitney *U* test. (5) Complication analysis: Incidences of acute confusion, sepsis, and reintubation postintervention were analyzed using the chi-square or Fisher's exact test. (6) The comparison of types of oxygen support before and after applying nursing programs was analyzed using Fisher's exact test.

## 10. Validity, Reliability, and Rigor

The program was developed based on a systematic review conducted by Worraphan et al. [21]. Once the program was developed, its validity and consistency with the program's objectives were evaluated by five multidisciplinary experts. Then, the program was modified based on the experts' recommendations, and it was tested on five older adults in the SICU to assess its feasibility. In addition, nurses and nursing assistants in the ward were trained to collaborate in providing care and promoting the patients' ability to perform ADLs as outlined in the program. Before beginning data collection, the research team registered the study guidelines in the Thai Clinical Trials Registry (TCTR) and adhered strictly to these guidelines throughout the research process. During data collection from the control and experimental groups, the research assistants, who were experienced and well-trained in outcome assessment, conducted the evaluations. The assessors were independent of the ward and unaware of whether the patients were assigned to the experimental or control groups. Moreover, the research team created a checklist to monitor and supervise the delivery of activities, ensuring that the older adults received all the activities outlined in the program to confirm that the results were attributable to the program.

## 11. Results

### 11.1. Demographic Information

This study included 50 participants, with no dropouts reported. The participants had a mean age of 72.12 ± 0.86 years, with the majority being female (52%). Most of them had worked in agriculture before retirement (66%). The primary reason for admission to the ICU was respiratory issues (56%). Hypertension was the most common underlying condition (68%). The APACHE II score was 19.92 ± 0.38 points. The malnutrition assessment showed that most participants were severely malnourished (NAF = C) (80%). Finally, no statistically significant differences were observed in demographic characteristics between the control and experimental groups (*p* > 0.05). Similarly, baseline comparisons of the primary outcomes showed no significant differences between the two groups (*p* > 0.05) (Table 1).

### 11.2. The Ability to Perform ADLs Within the Group

The comparison of ADL scores before and after the experiment (within-group comparison) showed that the control group had a mean score of 1.68 ± 1.15 before the experiment and 2.76 ± 0.44 after the experiment, which increased significantly (*p* < 0.01). In addition, the experimental group had a mean score of 1.84 ± 1.14 before the experiment and 17.48 ± 1.19 after the experiment, which increased significantly (*p* < 0.01) (Table 2).

### 11.3. The Ability to Perform ADLs Between Groups

The comparison of ADL scores between the control group and the experimental group (between-group comparison) showed that before the experiment, there was no significant difference in the ability to perform ADLs (*p*=0.57). However, after the experiment, the experimental group showed significantly higher scores in the ability to perform ADLs compared to the control group (*p* < 0.001) (Table 3).

### 11.4. Motor Power

The results of the motor power assessment before the experiment showed no significant difference in arms and legs motor power scores between the control group and the experimental group (*p*=0.56, *p*=0.74). However, after the experiment, the experimental group exhibited significantly higher motor power scores in both arms and legs than the control group (*p* < 0.001) (Table 4).

### 11.5. Complications

The experimental group demonstrated a significantly lower incidence of complications compared to the control group (*p* < 0.001). Specifically, the odds of experiencing any complication, delirium, sepsis, and reintubation were reduced in the experimental group, with odds ratios of 0.04, 0.03, 0.34, and 0.23, respectively (Table 5). In addition, after receiving the program, 64% of the experimental group could breathe without an oxygen cannula (room air), which was significantly higher than the control group (12%) (Table 6).

## 12. Discussion

The present study revealed that the group receiving the nursing program had significantly higher Barthel ADL Index scores than those receiving standard nursing care (*p* < 0.001). This result suggests that lung function, which is responsible for gas exchange and increasing oxygen in the bloodstream to nourish various body parts [22], plays a key role. When there is an obstruction or inability to exchange gases, older adults may experience fatigue, exhaustion, and a reduced desire to engage in activities. Therefore, the program developed in this study began by focusing on the lungs. It started with a deep breathing exercise performed in bed, a gentle activity that requires minimal effort but allows for full gas exchange by increasing the surface area for gas exchange and prolonging the time air stays in the lungs [23, 24]. An effective coughing exercise was also incorporated to help the older adults clear sputum more efficiently, enhancing the airways and increasing the surface area for gas exchange [25]. The program started with in-bed and bedside exercises. Training older adults to move quickly will allow them to breathe independently without needing an oxygen cannula more rapidly. Once older adults have no respiratory issues or are in a stable condition, they will have the strength to perform activities effectively. The present study observed that 64% of the group that received the program could breathe without an oxygen cannula, while 36% still required an oxygen cannula. In contrast, only 12% of the control group were able to breathe without an oxygen cannula, 68% required a HFNC, and 12% needed to be re-intubated (*p* < 0.01). Therefore, rehabilitation and promoting the ability to perform ADLs in older adults in the ICU or SICU should begin with lung exercises. This approach enhances the efficiency of performing other activities and helps prevent subsequent complications.

Regarding the promotion of the feeding ability, after the experiment, the experimental group showed a significantly greater improvement in feeding ability scores than the control group (*p* < 0.01). Promoting the feeding ability in older adults must consider physical changes such as reduced taste buds, fewer teeth, decreased appetite, impaired chewing ability, reduced muscle strength, stiffness, and unfamiliarity with food and eating environments. These factors can lead to decreased access to food and difficulty eating for older adults [26]. Therefore, the program promoted feeding ability by incorporating limb exercises to increase strength and help older adults feed themselves [24]. It also included food preparation strategies, such as assessing for choking hazards and testing swallowing in older adults who recently had extubation [27]. The menu was also adjusted to suit the older adults by offering soft, easy-to-chew foods and considering their food preferences, as long as these preferences did not conflict with the treatment plan. Furthermore, sour tastes were added to enhance the function of the taste buds of older adults [26]. The taste and the duration of meals were also a concern, following the principle of “serve first, collect later” [28] to avoid rushing them while eating. Furthermore, their relatives were encouraged to stimulate the older adults to eat, and the environment was arranged to resemble a home setting to promote adequate food intake [29]. The results of the present study showed that the older adults in the experimental group did not experience choking on food and were able to feed themselves. This result indicates that promoting the feeding ability in older adults requires considering their aging change processes and adjusting interventions to fit their characteristics and the ward environment. Such approaches will enable older adults to regain independence, rehabilitate their bodies, and, upon returning home, be able to care for themselves without relying on others.

For promoting the grooming ability, we found that after the experiment, the grooming scores were significantly higher than before the experiment (*p*=0.01). The changes observed in the older adults included a decrease in the number of teeth, a lack of knowledge, understanding, and skills in brushing teeth appropriately, as well as health beliefs such as the misconception that using a hard and large toothbrush leads to cleaner teeth [30]. The program developed an innovative toothbrush box for older adults, featuring a small, soft-bristled brush to accommodate the low platelet count common in ICU patients, helping to prevent bleeding gums and reduce oral injuries [31]. The box also included Vaseline to moisturize the lips, promoting a healthier appearance. The program also provided other supportive equipment, such as a hair comb, mirror, and shaver, to make it easier for older adults to care for themselves. This approach can reduce unnecessary energy expenditure for searching, preparing, and storing grooming tools. As a result, older adults can wash their faces, brush their teeth, comb their hair, and shave independently. Moreover, they expressed high satisfaction with the box, as evidenced by the feedback reflecting the positive outcomes of using the developed program, as follows:

“The experimental group was satisfied with the program, as the toothbrush was the right size, did not irritate the mouth, and did not cause bleeding while brushing. In addition, it was easy to use, saving time and energy that would otherwise be spent on preparation or searching for the tools.” (PT0001)

“The experimental group expressed high satisfaction with the program. It helped promote their recovery once the crisis had passed, enabling them to engage in exercises and activities instead of merely lying idle and feeling bored. They also appreciated the care and attention they received and expressed a desire for nurses to implement this approach with all patients. Moreover, the involvement of relatives in providing care and encouragement was greatly valued.” (PT0002)

“The registered nurses were satisfied with the program because it encouraged the older adults to wash their faces and brush their teeth independently. The equipment provided was well-suited for the older adults, contributing to the patients' healthier appearance after the crisis.” (NU0001)

For promoting the transfer ability, after the experiment, we found that the transfer ability was significantly improved compared to before the experiment (*p* < 0.01). This improvement occurred because the older adults had just recovered from their critical conditions, during which their motor power had decreased, muscles had atrophied, and they had been inactive for an extended period. The program encouraged them to begin exercising after the crisis. Nurses assessed the stability of the patient's vital signs before initiating exercise [21]. They started with in-bed exercises, including active range of motion (AROM), passive range of motion (PROM), and muscle contractions in the arms and legs to increase strength [24, 32]. These bed exercises helped prepare the joints and muscles for movement, preventing stiffness. Once the older adults' motor power improved, the next step involved bedside exercises, resistance training, muscle contractions [3], and bedside walking exercises. These allowed the older adults to move more quickly, reduce bedridden time [32], and assist them in performing daily activities more independently. In addition, encouraging their relatives to support the older adults' exercise routines enhanced their performance. The older adults were more comfortable with their relative's manners, voices, and familiar language. With set goals and consistent encouragement from family members, older adults tended to recover more quickly [29]. This finding underscores the importance of exercise programs in empowering older adults to maintain independence and reduce reliance on others. In addition, these programs promote physical and mental strength, enhance motivation, provide social support, facilitate patient education, and encourage family engagement, all of which contribute to recovery, functional improvement, and readiness for returning home.

In the case of promoting the bowel, bladder, toileting, bathing, and dressing abilities, we found that after the experiment, the scores were significantly higher than before the experiment (*p* < 0.01). This improvement occurred because older adults often experienced a decline in bowel and bladder control and mobility [33]. Moreover, in the ICU, prolonged use of urinary catheters and disposable diapers often leads to complications such as incontinence-associated dermatitis and pressure sores [34]. These factors led the older adults to believe they could not defecate, dress, or bathe independently. Therefore, this program included activities designed to encourage them to regain independence after their critical conditions had passed. It involved removing the urinary catheter when there was no indication [29], encouraging them to use the bathroom independently, and preparing equipment for defecation near their beds for convenience. The program also encouraged the older adults to bathe, dress, and care for their hygiene independently, with support from their relatives and nurses providing close assistance. Involving relatives in these activities can help reduce older adults' embarrassment. Promoting the bowel, bladder, toileting, bathing, and dressing abilities in older adults requires scientific knowledge and compassionate care to ensure success. The improvements observed in this study are likely attributable to the appropriate ward context and study program, including ward structure, patient conditions (e.g., step-down or partial recovery), nurse-to-patient ratio, and the presence of family support during admission. These factors should be considered for future program implementation.

The results of the complication assessment indicated that the experimental group experienced significantly fewer complications than the control group (*p* < 0.01). When assessing acute confusion using the confusion assessment method (CAM), it was found that 40% of the control group experienced acute confusion, whereas no cases of acute confusion occurred in the experimental group. This finding can be attributed to the stress and anxiety that older adults in the ICU/SICU often face due to critical conditions, unfamiliar surroundings, and people, all of which can trigger acute confusion [35]. The program mitigated risk factors for acute confusion in older adults by promoting lung function to ensure adequate oxygen supply to the brain and other body parts, encouraging daytime activities, facilitating nighttime sleep, engaging in physical exercises, involving relatives in the program, and promoting regular urination before bedtime [35]. These approaches can reduce risk factors and help prevent complications, particularly acute confusion.

The results of the motor power assessment showed that the experimental group had significantly greater motor power than the control group (*p* < 0.01). Older adults admitted to the ICU often experience prolonged immobility, along with malnutrition, leading to reduced motor power, weakened limbs, and muscle atrophy [4]. The program for the experimental group included a series of exercises, starting with in-bed exercises focusing on lung preparation through breathing and coughing techniques. It also incorporated bedside exercises and bedside walking. In addition, a protein-rich diet was introduced to promote muscle protein synthesis, which is particularly beneficial for bedridden patients with limited mobility to help recover their motor power [36]. Furthermore, nurses encouraged older adults to exercise and engage in rehabilitation activities, helping them recover motor power more effectively after leaving the ICU than those who did not participate in the developed program.

## 13. Limitations

The nursing program to promote the ADLs after passing critical conditions among older adults was implemented over a relatively short duration of 7 days, which limited the ability to assess all variables, such as stairs and the overall quality of life of the older adults. Some results, including ADL, motor power, and complication, report very large differences between groups. The possibility of measurement bias or the Hawthorne effect might be the cause of the difference. While intraobserver reliability was high (0.97), interobserver reliability was lower (0.71), indicating a limitation in the consistency of the outcome measurement. Moreover, the program was tailored to fit the specific service context of the ward, which may present limitations for its application in other wards or healthcare settings.

## 14. Conclusion and Recommendations

### 14.1. Conclusion

This study examined the effects of a nursing program on promoting early ADLs after passing critical conditions among older adults. The experimental group that received the program demonstrated a significantly better ability to perform ADLs than the control group, which received standard nursing care. In addition, the developed program effectively reduced complications such as acute confusion, improved lung function, reduced oxygen cannula use, and increased motor power among older adults.

### 14.2. Recommendations

Extending the program duration and systematically evaluating outcomes could further enhance effectiveness and provide robust evidence for the long-term sustainability of the observed benefits. The program's long-term effects on promoting the ability to perform ADLs among older adults should be investigated over periods of 1,3, and 6 months. In addition, the program should be expanded to the ICUs that treat specific conditions, such as stroke, heart disease, or musculoskeletal injuries, with adjustments made to suit the context of different wards. A more rigorous research design, such as a randomized controlled trial (RCT) with blinding of participants and nurses, is recommended to validate these findings and enhance their generalizability, as the quasi-experimental design with nonrandomized allocation limits internal validity and introduces potential selection bias. In addition, future studies should explore other relevant variables, including stair negotiation, quality of life, mental health, and caregiver burden.

## Figures and Tables

**Table 1:** General information of older patients.

General information	All samples (*n* = 50)		Control (*n* = 25)		Experimental (*n* = 25)		*p* value
	*n*	%	*n*	%	*n*	%	
Sex							0.571^C^
Female	26	52.00	12	48.00	14	56.00	
Male	24	48.00	13	52.00	11	44.00	
Age (years)							0.581^C^
60–69 years	21	42.00	11	44.00	10	40.00	
70–79 years	25	50.00	13	52.00	12	48.00	
80 years and over	4	8.00	1	4.00	3	12.00	
Min–Max (mean ± S.D.)	60–87 (72.12 ± 0.86)	61–82 (72.28 ± 1.15)	60–87 (71.96 ± 1.29)	0.818^I^			
Body mass index (kg/m^2^)							0.252^F^
Less than 18.50	6	12.00	5	20.00	1	4.00	
18.50–22.99	31	62.00	14	56.00	17	68.00	
More than 23.00	13	26.00	6	24.00	7	28.00	
Min–Max (mean ± S.D.)	15.57–30.22 (21.24 ± 0.43)	15.57–27.24 (20.69 ± 0.54)	17.35–30.22 (21.78 ± 0.66)	0.268^I^			
Occupation							0.376^F^
Unemployed	33	66.00	18	72.00	15	60.00	
Government employee	9	18.00	4	16.00	5	20.00	
Self-employed	8	16.00	3	12.00	5	12.00	
Cause of admission							0.888^F^
Respiratory disease	28	56.00	15	60.00	13	52.00	
Cardiovascular disease	11	22.00	5	20.00	6	24.00	
Gastrointestinal and liver disease	8	16.00	3	12.00	5	20.00	
Urinary tract	2	4.00	1	4.00	1	4.00	
Other	1	2.00	1	4.00			
APACHE II score							0.382^C^
0–19 score	19	38.00	8	32.00	11	44.00	
More than 19 score (severe level of the disease)	31	62.00	17	68.00	14	56.00	
Min–Max (mean ± S.D.)	10–25 (19.92 ± 0.38)	16–24 (20.44 ± 0.40)	10–25 (19.40 ± 0.63)	0.187^I^			
Congenital/chronic diseases (more than 1 answer can be given)							
Hypertension	34	68.00	18	72.00	16	64.00	0.287^F^
Diabetes	28	56.00	15	60.00	13	52.00	0.378^F^
Hyperlipidemia	30	60.00	15	60.00	15	60.00	0.167^F^
Heart disease	11	22.00	5	20.00	6	24.00	0.301^F^
Respiratory disease	18	36.00	10	40.00	8	32.00	0.780^F^
Kidney disease	11	22.00	5	20.00	6	24.00	0.390^F^
Cancer	6	12.00	2	8.00	4	16.00	0.271^F^
Cirrhosis	7	14.00	3	12.00	4	16.00	0.252^F^
Others	6	12.00	3	12.00	3	12.00	0.300^F^
Risk of malnutrition (SPENT)							
High risk	50	100.00	25	100.00	25	100.00	0.00
Nutrition status (NAF)							0.138^F^
Moderate malnutrition	10	20.00	7	28.00	3	12.00	
Severe malnutrition	40	80.00	18	72.00	22	88.00	
Min–Max (mean ± S.D.)	8–17 (12.68 ± 0.31)	8–24 (12.20 ± 0.47)	10–17 (13.16 ± 0.38)	0.378^I^			

Abbreviations: NAF = Nutrition Alert Form; S.D. = standard deviation; SPENT = Society of Parenteral and Enteral Nutrition of Thailand.

^I^Independent *t*-test.

^C^Chi-square test.

^F^Fisher's exact test.

**Table 2:** The comparison of the Barthel Index for activities of daily living (ADLs) before and after applying the nursing program to early promote the activities of daily living among older patients (within-group comparison).

Outcomes	Groups	Before			After			Mean rank	Sum of rank	*Z*	*p* value
		Median	Mean	SD	Median	Mean	SD				
Total Barthel ADL	Control	2.00	1.68	1.15	3.00	2.76	0.44	9.00	153.00	−3.72	< 0.01
	Experimental	2.00	1.84	1.14	18.00	17.48	1.19	13.00	325.00	−4.39	< 0.01

Feeding	Control	0.00	0.24	0.44	1.00	0.92	0.23	9.00	153.00	−4.12	< 0.01
	Experimental	0.00	0.32	0.48	2.00	1.88	0.33	12.50	300.00	−4.44	< 0.01

Grooming	Control	1.00	0.64	0.49	1.00	1.00	0.00	5.00	45.00	−3.00	0.003
	Experimental	1.00	0.76	0.44	2.00	1.00	0	3.50	21.00	−2.45	0.01

Transfers (bed to chair and back)	Control	0.00	0.28	0.46	1.00	1.00	0.41	8.50	136.00	−3.82	< 0.01
	Experimental	1.00	0.67	0.44	2.00	2.48	0.51	13.00	325.00	−4.45	< 0.01

Toilet use	Control	1.00	1.00	0.41	0.00	0.00	0.00	0.00	0.00	0.00	1.00
	Experimental	0.00	0.00	0.00	2.00	2.36	0.57	13.00	325.00	−4.50	< 0.01

Mobility on level surfaces	Control	0.00	0.00	0.00	0.00	0.00	0.00	0.00	0.00	0.00	1.00
	Experimental	0.00	0.00	0.00	2.00	2.00	0.00	13.00	325.00	−5.00	< 0.01

Dressing	Control	0.00	0.00	0.00	0.00	0.00	0.00	0.00	0.00	0.00	1.00
	Experimental	0.00	0.00	0.00	2.00	1.84	0.37	13.00	325.00	−4.72	< 0.01

Stairs	Control	0.00	0.00	0.00	0.00	0.00	0.00	0.00	0.00	0.00	1.00
	Experimental	0.00	0.00	0.00	0.00	0.84	0.37	6.00	66.00	−3.32	< 0.01

Bathing	Control	0.00	0.00	0.00	0.00	0.00	0.00	0.00	0.00	0.00	1.00
	Experimental	0.00	0.00	0.00	1.00	0.84	0.37	11.00	213.00	−4.58	< 0.01

Bowel control	Control	0.00	0.00	0.00	0.00	0.00	0.00	0.00	0.00	0.00	1.00
	Experimental	0.00	0.00	0.00	2.00	1.80	0.41	13.00	325.00	−4.67	< 0.01

Bladder control	Control	0.00	0.00	0.00	0.00	0.00	0.00	0.00	0.00	0.00	1.00
	Experimental	0.00	0.00	0.00	3.00	2.84	0.37	13.00	325.00	−4.72	< 0.01

*Note:* Wilcoxon signed rank test.

Abbreviations: ADL = activities of daily living; S.D. = standard deviation.

**Table 3:** The comparison of the Barthel Index for Activities of Daily Living (ADLs) before and after applying the nursing program to early promote the activities of daily living among older patients (between-group comparison).

Outcome	Measured time	Control					Experimental					Mann–Whitney *U* test	*Z*	*p* value
		Median	Mean	SD	Mean rank	Sum of ranks	Median	Mean	SD	Mean rank	Sum of ranks			
Total Barthel ADL	Before	2.00	1.68	1.15	24.40	610.00	2.00	1.84	1.14	26.60	665.00	285.00	−0.57	0.57
	After	3.00	2.76	0.44	13.00	325.00	18.00	17.48	1.19	38.00	950.00	0.00	−6.28	< 0.01

Feeding	Before	0.00	0.24	0.44	24.50	621.00	0.00	0.32	0.48	26.50	662.50	287.50	−0.62	0.53
	After	1.00	0.92	0.23	14.38	359.50	2.00	1.88	0.33	36.62	915.50	34.50	−6.13	< 0.01

Grooming	Before	1.00	0.64	0.49	24.00	600.00	1.00	0.76	0.44	27.00	675.00	275.00	−0.92	0.36
	After	1.00	1.00	0.00	25.50	637.50	2.00	1.00	0	25.50	637.50	312.50	0.00	1.00

Transfers (bed to chair and back)	Before	0.00	0.28	0.46	19.50	487.50	1.00	0.67	0.44	31.50	787.50	162.50	−3.36	0.34
	After	1.00	1.00	0.41	13.52	338.00	2.00	2.48	0.51	37.48	937.00	13.00	−6.18	< 0.01

Toilet use	Before	1.00	1.00	0.41	25.50	637.50	0.00	0.00	0.00	25.50	637.50	312.50	0.00	1.00
	After	0.00	0.00	0.00	13.00	325.00	2.00	2.36	0.57	38.00	950.00	0.00	−6.60	< 0.01

Mobility on level surfaces	Before	0.00	0.00	0.00	25.50	637.50	0.00	0.00	0.00	25.50	637.50	312.50	0.00	1.00
	After	0.00	0.00	0.00	13.00	325.00	2.00	2.00	0.00	38.00	950.00	0.00	−7.00	< 0.01

Dressing	Before	0.00	0.00	0.00	25.50	637.50	0.00	0.00	0.00	25.50	637.50	312.50	0.00	1.00
	After	0.00	0.00	0.00	13.00	325.00	2.00	1.84	0.37	38.00	950.00	0.00	−6.78	< 0.01

Stairs	Before	0.00	0.00	0.00	25.50	637.50	0.00	0.00	0.00	25.50	637.50	312.50	0.00	1.00
	After	0.00	0.00	0.00	20.00	500.00	0.00	0.84	0.37	31.00	775.00	175.00	−3.72	< 0.01

Bathing	Before	0.00	0.00	0.00	25.50	637.50	0.00	0.00	0.00	25.50	637.50	312.50	0.00	1.00
	After	0.00	0.00	0.00	15.00	375.00	1.00	0.84	0.37	36.00	900.00	50.00	−5.96	< 0.01

Bowel control	Before	0.00	0.00	0.00	25.50	637.50	0.00	0.00	0.00	25.50	637.50	312.50	0.00	1.00
	After	0.00	0.00	0.00	13.00	325.00	2.00	1.80	0.41	38.00	950.00	0.00	−6.74	< 0.01

Bladder control	Before	0.00	0.00	0.00	25.50	637.50	0.00	0.00	0.00	25.50	637.50	312.50	0.00	1.00
	After	0.00	0.00	0.00	13.00	325.00	3.00	2.84	0.37	38.00	950.00	0.00	−6.78	< 0.01

*Note:* Mann–Whitney *U* test.

Abbreviations: ADL = activities of daily living; S.D. = standard deviation.

**Table 4:** The comparison of motor power before and after applying nursing programs to early promote the activities of daily living among older patients (between-group comparison).

Motor power	Measured time	Control (*n* = 25)					Experimental (*n* = 25)					Mann–Whitney *U* test	*Z*	*p* value
		Median	Mean	SD	Mean rank	Sum of ranks	Median	Mean	SD	Mean rank	Sum of ranks			
Right arm	Before	3.00	3.40	0.50	26.50	662.50	3.00	3.32	0.48	24.50	612.50	287.50	−0.58	0.56
	After	4.00	3.68	0.48	13.34	333.50	5.00	4.96	0.20	37.66	941.50	8.50	−6.44	< 0.001

Left arm	Before	3.00	3.40	0.50	26.50	662.50	3.00	3.32	0.48	24.50	612.50	287.50	−0.58	0.56
	After	4.00	3.68	0.48	13.34	333.50	5.00	4.96	0.20	37.66	941.50	8.50	−6.44	< 0.001

Right leg	Before	3.00	3.24	0.36	26.00	650.00	3.00	3.20	0.41	25.00	625.00	300	−0.34	0.74
	After	4.00	3.60	0.50	13.60	340.00	5.00	4.92	0.28	37.40	935.00	15.00	−6.24	< 0.001

Left leg	Before	3.00	3.24	0.36	26.00	650.00	3.00	3.20	0.41	25.00	625.00	300	−0.34	0.74
	After	4.00	3.60	0.50	13.60	340.00	5.00	4.92	0.28	37.40	935.00	15.00	−6.24	< 0.001

*Note:* Mann–Whitney *U* test.

Abbreviation: S.D. = standard deviation.

**Table 5:** The comparison of complications after applying nursing programs to early promote the activities of daily living among older patients (between-group comparison).

Outcome		Control (*n* = 25)		Experimental (*n* = 25)		*x* ^2^	*p* value	Odd ratio
		*n*	%	*n*	%			
Total complication	No	8	32.00	23	92.00	19.10	< 0.01^C^	0.04
	Yes	17	68.00	2	8.00			

Delirium	No	15	60.00	25	100.00	12.50	< 0.01^F^	0.03
	Yes	10	40.00	0	0			

Sepsis	No	16	64.00	21	84.00	2.59	0.107^C^	0.34
	Yes	9	36.00	4	16.00			

Reintubation	No	23	92.00	25	100.00	0.347	0.769^F^	0.23
	Yes	2	8.00	0	0			

^C^Chi-square test.

^F^Fisher's exact test.

**Table 6:** The comparison of oxygen support used before and after applying nursing programs to early promote the activities of daily living among older patients (between-group comparison).

Outcome	Type oxygen	Before				*x* ^2^	*p* value	After				*x* ^2^	*p* value
		Control (*n* = 25)		Experimental (*n* = 25)				Control (*n* = 25)		Experimental (*n* = 25)			
		*n*	%	*n*	%			*n*	%	*n*	%		
Oxygen, suppose used	Room air	0	0	0	0	1.09	1.00^F^	3	12.00	16	64.00	33.35	< 0.01^F^
	Cannula	1	4.00	0	0			2	8.00	9	36.00		
	HFNC	20	80.00	20	80.00			17	68.00	0	0		
	NIV	4	16.00	5	20.00			0	0	0	0		
	ET-tube	0	0	0	0			3	12.00	0	0		

Abbreviations: ET-tube = endotracheal tube; HFNC = high-flow nasal cannula; NIV = noninvasive ventilator.

^F^Fisher's exact test.

## Data Availability

The datasets used and/or analyzed during the current study are available from the corresponding author upon reasonable request.

## References

[1] Guidet B., Vallet H., Boddaert J. (2018). Caring for the Critically Ill Patients over 80: A Narrative Review. *Annals of Intensive Care*.

[2] Zhang L., Hu W., Cai Z. (2019). Early Mobilization of Critically Ill Patients in the Intensive Care Unit: A Systematic Review and Meta-Analysis. *PLoS One*.

[3] Phasook N., Ua-Kit N. (2015). The Effect of Early Mobilization Program on Duration of Mechanical Ventilation in Critically Ill Medical Patients. *Thai Journal of Cardio-Thoracic Nursing*.

[4] Tymkew H., Norris T., Arroyo C., Schallom M. (2020). The Use of Physical Therapy ICU Assessments to Predict Discharge Home. *Critical Care Medicine*.

[5] Matsumoto T., Yoshikawa R., Harada R. (2023). Predictors of Activities of Daily Living in Intensive Care Unit Survivors: A Propensity Score Matching Analysis. *Progress in Rehabilitation Medicine*.

[6] Ding X., Lian H., Wang X. (2021). Management of Very Old Patients in Intensive Care Units. *Aging and disease*.

[7] Shaban M., Elsayed Ramadan O. M., Zaky M. E., Mohamed Abdallah H. M., Mohammed H. H., Abdelgawad M. E. (2025). Enhancing Nursing Practices in Critical Care for Older Adults: A Systematic Review of Age-Friendly Nursing Interventions. *Journal of the American Medical Directors Association*.

[8] Brunker L. B., Boncyk C. S., Rengel K. F., Hughes C. G. (2023). Elderly Patients and Management in Intensive Care Units (ICU): Clinical Challenges. *Clinical Interventions in Aging*.

[9] Dantas C. M., Silva P. F., Siqueira F. H. (2012). Influence of Early Mobilization on Respiratory and Peripheral Muscle Strength in Critically Ill Patients. *Revista Brasileira de Terapia Intensiva*.

[10] Doiron K. A., Hoffmann T. C., Beller E. M. (2018). Early Intervention (Mobilization or Active Exercise) for Critically Ill Adults in the Intensive Care Unit. *Cochrane Database of Systematic Reviews*.

[11] Pashmdarfard M., Azad A. (2020). Assessment Tools to Evaluate Activities of Daily Living (ADL) and Instrumental Activities of Daily Living (IADL) in Older Adults: A Systematic Review. *Medical Journal of the Islamic Republic of Iran*.

[12] Hunter E. G., Kearney P. J. (2018). Occupational Therapy Interventions to Improve Performance of Instrumental Activities of Daily Living for Community-Dwelling Older Adults: A Systematic Review. *American Journal of Occupational Therapy*.

[13] Li X., Zheng T., Guan Y. (2020). ADL Recovery Trajectory After Discharge and Its Predictors Among Baseline-Independent Older Inpatients. *BMC Geriatrics*.

[14] Buurman B. M., Han L., Murphy T. E. (2016). Trajectories of Disability Among Older Persons Before and After a Hospitalization Leading to a Skilled Nursing Facility Admission. *Journal of the American Medical Directors Association*.

[15] Che Y. J., Qian Z., Chen Q., Chang R., Xie X., Hao Y. F. (2023). Effects of Rehabilitation Therapy Based on Exercise Prescription on Motor Function and Complications After Hip Fracture Surgery in Elderly Patients. *BMC Musculoskeletal Disorders*.

[16] Simim S., Banharak S., Panpanit L. (2024). Translation, Psychometric Testing and Implementation of the Perineal Assessment Tool for Assessing incontinence-Associated Dermatitis Risk in Semi-Intensive Care Patients. *Scientific Reports*.

[17] Fleiss J. L., Levin B., Paik M. C. (2003). *Statistical Methods for Rates and Proportions*.

[18] Ichikawa T., Tsuchiya A., Tsutsumi Y. (2025). Effect of a Generalized Early Mobilization and Rehabilitation Protocol on Outcomes in Trauma Patients Admitted to the Intensive Care Unit: A Retrospective Pre-Post Study. *Critical Care*.

[19] Grove S. K., Burns N., Gray J. R. (2013). *The Practice of Nursing Research, Appraisal, Synthesis and Generation of Evidence*.

[20] Loahaprasitiporn P., Jarusriwanna A., Unnanuntana A. (2017). Validity and Reliability of the Thai Version of the Barthel Index for Elderly Patients with Femoral Neck Fracture. *Journal of the Medical Association of Thailand*.

[21] Worraphan S., Thammata A., Chittawatanarat K., Saokaew S., Kengkla K., Prasannarong M. (2020). Effects of Inspiratory Muscle Training and Early Mobilization on Weaning of Mechanical Ventilation: A Systematic Review and Network Meta-Analysis. *Archives of Physical Medicine and Rehabilitation*.

[22] Brinkman J. E., Sharma S. (2023). Physiology, Pulmonary. *StatPearls*.

[23] Stiller K K. (2013). Physiotherapy in Intensive Care: An Updated Systematic Review. *Chest*.

[24] Kayambu G., Boots R., Paratz J. (2015). Early Physical Rehabilitation in Intensive Care Patients With Sepsis Syndromes: A Pilot Randomised Controlled Trial. *Intensive Care Medicine*.

[25] Stiller K. (2013). Physiotherapy in Intensive Care: An Updated Systematic Review. *Chest*.

[26] Thompson L. A., Chen H. (2021). Physiology of Aging of Older Adults: Systemic and Oral Health Considerations-2021 Update. *Dental Clinics of North America*.

[27] Christmas C., Rogus‐Pulia N. (2019). Swallowing Disorders in the Older Population. *Journal of the American Geriatrics Society*.

[28] Limpawattana P., Phimson K., Sookprasert A., Sirithanaphol W., Chindaprasirt J. (2020). Prevalence of Geriatric Syndromes in Elderly Cancer Patients Receiving Chemotherapy. *Current gerontology and geriatrics research*.

[29] Davidson J. E., Harvey M. A., Bemis-Dougherty A., Smith J. M., Hopkins R. O. (2013). Implementation of the Pain, Agitation, and Delirium Clinical Practice Guidelines and Promoting Patient Mobility to Prevent Post-Intensive Care Syndrome. *Critical Care Medicine*.

[30] Janto M., Iurcov R., Daina C. M. (2022). Oral Health Among Elderly, Impact on Life Quality, Access of Elderly Patients to Oral Health Services and Methods to Improve Oral Health: A Narrative Review. *Journal of Personalized Medicine*.

[31] Sonpanao P. (2019). Oral Health Care for the Elderly. *Journal of Health Research and Development Nakhon Ratchasima Provincial Public Health Office*.

[32] Hodgson C. L., Bailey M., Bellomo R. (2016). A Binational Multicenter Pilot Feasibility Randomized Controlled Trial of Early Goal-Directed Mobilization in the ICU. *Critical Care Medicine*.

[33] Davis N. J., Wyman J. F., Gubitosa S., Pretty L. (2020). Urinary Incontinence in Older Adults. *American Journal of Nursing*.

[34] Banharak S., Panpanit L., Subindee S. (2021). Prevention and Care for Incontinence-Associated Dermatitis Among Older Adults: A Systematic Review. *Journal of Multidisciplinary Healthcare*.

[35] Seyffert S., Moiz S., Coghlan M. (2022). Decreasing Delirium Through Music Listening (DDM) in Critically Ill, Mechanically Ventilated Older Adults in the Intensive Care Unit: A Two-Arm, Parallel-Group, Randomized Clinical Trial. *Trials*.

[36] Vanhorebeek I., Latronico N., Van den Berghe G. (2020). ICU-Acquired Weakness. *Intensive Care Medicine*.

